# Galvanic Action of Metals in the Mouth

**Published:** 1840

**Authors:** Josephus Brockway


*For the American Journal of Dental Science.*

### GALVANIC ACTION OF METALS IN THE MOUTH.

—

[ BY JOSEPHUS BROCKWAY. ]

Messrs. Editors :—

Careful observation has satisfied me, that the present method of inserting teeth on gold plate, ought not to be continued ; the different metals combined, convert the mouth into a galvanic battery.

It is not my purpose, were I competent, to discuss philosophically the " modus operandi" of galvanic action. I wish, simply to state a few plain facts connected with the practice of dentistry, leaving to the learned and practical chemist, the developement of just theory ; simply premising, that galvanism is defined as being the method of exciting the electric principle by the action of some solutions upon two or more metals, differing in degrees of affinity for oxygen. It is a fact, the knowledge of which, is as old as galvanism itself—that the human saliva affords a solution fully competent to call into action the electric principle. Any two metals differing in degrees of attraction for oxygen, put into the mouth, produce at once a sensation and taste peculiar to galvanic action.

Deeming it unnecessary further to pursue the general principle, I come at once to a statement of facts, showing the inexpediency of continuing the present method of inserting teeth upon gold plate. It will therefore, be necessary, first to state what I suppose to be the method now in common use.

The practice which now prevails, and for several years has prevailed, both in Europe and America, is attended with the introduction of from three to six different metals into the same mouth.

First.—The mineral teeth have platina pins made fast to them in their fabrication.

Second.—These pins are attached to alloyed gold. The gold generally used being eighteen parts fine gold, and six silver and copper in different proportions, making eighteen carat gold.

Third.—The teeth are fastened to this alloyed gold by a still baser alloy of gold, called gold solder. This is usually made one half gold and the other half silver and copper, with the addition, frequently, or substitution, of brass or speltre—thus making the solder to consist of gold, silver, copper, and zinc. Some, indeed, use their solder and gold in greater purity, but none avoid the use of four different metals, differing in degrees of attraction for oxygen. I will name them in their order of affinity. Zinc, when used, copper, silver, gold, platina ; or as the metals present themselves, ready for use, to the eye of the dentist, they stand in order, solder, gold, platina—and to these is often added, in the same mouth, tin—some of the teeth being filled with this metal, not to mention the poisonous metals, such as bismuth, and mercury, which quackery has introduced into the mouth, in the form of amalgams, fusible metals, pastes, succedaneums, &c.

The infancy of knowledge on the subject of galvanic action, ought, indeed, to offer an apology for the past usage of scientific dentists; but such will look in vain for a covert from the charge of quackery, if they refuse to submit to the lights which science and facts are daily disclosing on this subject.

I therefore proceed with my facts:—

And first,—I will state a single case as illustrative of the effects of two metals used in connexion, in the same mouth, upon the nervous system. About ten years ago, I inserted ten teeth for a Rev. gentleman, in Albany, New-York. This was previous to the common use of mineral teeth. I used the animal; they were united by a large flattened gold wire passing through the whole, turned at the ends, forming clasps or hooks, connecting them with the molar teeth; several of the teeth thus strung together, were hollow, that is, the cavities formerly occupied by the nerve and blood vessels were open. These cavities I filled with *tin foil.* This foil was thus brought in contact with the gold. On putting these teeth, thus prepared, into the mouth, the patient received several successive shocks, exclaiming, "what's that?" "what's that?" I told him I suspected I had erected a galvanic battery in his mouth, and immediately took them out and substituted gold instead of the tin,—and thus remedied the evil. But although this fact arrested me in the use of tin and gold in such a connexion, I did not then perceive the impropriety of using tin and gold in the same mouth, for I had the mistaken impression, that the two metals must come in contact in order to produce galvanic action.

But this fact has kept me somewhat awake to this subject, although, I confess I have been slow to admit the true cause of an almost every day complaint of patients. I should think it had been the fact in one half the cases where I have used such plates as described above, that I heard from my patient, among the first exclamations, "what makes this taste so?"—"Why how this tastes!"—"It tastes brassy!" &c. &c. For a long time I explained, and honestly too, by stating to them the fact that it had been cleansed in acid, and was not free from the taste of it, but soon would be. But since my attention has been called to the subject, I find it a fact too obvious to meet with a denial, that the plates thus combined, have a strong galvanic taste and action, like all base compound and combined metals; a taste and action upon the nerves, which only becomes tolerable by becoming familiar. But how far this galvanic taste and action is injurious to the nervous system, I pretend not to judge.

But I come now to what I conceive to be a palpable and deleterious effect. The galvanic action thus induced destroys the natural teeth.

The effects upon both the teeth and the nerves, differ, of course, with the difference in the nervous temperament, and the condition of the salivary secretion. Hence the injury sustained by teeth in contact with these plates will be much more apparent in some cases, than in others. But I doubt not every dentist who has carefully watched the results of his own operations, has been occasionally mortified and grieved on seeing the waste which has evidently followed the application of such plates.

But to this point I must bring a single case as an illustration. About twelve years ago, I set two lateral incisors, for Mr. H. of the city of Troy, N. Y. He was then, and now is, a man of sound constitution and good health, and had already passed the age in which the teeth of males are liable to the attacks of caries, so that it was not to be expected that his teeth, then sound, would fall the prey of this disease. The teeth which I set, at that time, were human teeth, set in a manner common with me. The two were connected by a flattened wire, as in the case before mentioned, and hooked to two neighboring teeth, of course no solder was used, nor other metal except gold.

These two teeth were worn about six years, during which time no symptoms of disease appeared about his other teeth, but when these were removed, now six years since, a gold plate with solder after the usual manner, was substituted. Since this, a most lamentable change has taken place in the condition of his teeth. They very soon began to exhibit the appearance of teeth, in a mouth surcharged with acidity, not unlike what is often seen in the case of a female during gestation, a strong and unconquerable disposition to decay, which it was found impossible to arrest by plugging, so that now instead of two, he is obliged to wear five artificial teeth, while several others have required plugging. I state this as being at once a strong and palpable case of galvanic action, resulting in the decomposition of teeth. I believe, however, it is a case which has parallels by the thousand.

The conviction, therefore, to me, is irresistible, that the use of compound and mixed metals in the mouth,is deleterious to the general health; but more especially to the health of the teeth; I therefore give it as my unhesitating opinion, that the present method of inserting the mineral teeth, must be superceded by a practice which shall allow of the use of but one metal in the same mouth; such a practice is attainable, and aside from the above considerations, will be attended with advantage, both to the dentist and his patients.

For nearly twenty years I have been in the habit of using gold hooks to fasten artificial teeth to natural ones; and when used unconnected with other metals, I have never known them occasion caries, though they sometimes wear, to their injury, the teeth to which they are attached. I conclude, therefore, that pure gold may be used without detriment, either within or around, the teeth, provided no other metal be used in the same mouth; and from long observation, I give it as my opinion, that, while it is objectionable to use gold in filling the front teeth, and tin for the back ones, the evil is very trifling, compared with the combination of metals as used at present in setting teeth in gold plates.

